# High-Value Components and Bioactives from Sea Cucumbers for Functional Foods—A Review

**DOI:** 10.3390/md9101761

**Published:** 2011-10-10

**Authors:** Sara Bordbar, Farooq Anwar, Nazamid Saari

**Affiliations:** 1 Faculty of Food Science and Technology, Universiti Putra Malaysia, Serdang, Selangor 43400, Malaysia; E-Mails: sara_bordbar@ymail.com (S.B.); fqanwar@yahoo.com (F.A.); 2 Department of Chemistry and Biochemistry, University of Agriculture, Faisalabad 38040, Pakistan

**Keywords:** sea cucumber bioactives, antioxidant nutrients, triterpene glycosides, glycosaminoglycan, functional peptides, biological activities, medicinal health functions

## Abstract

Sea cucumbers, belonging to the class *Holothuroidea*, are marine invertebrates, habitually found in the benthic areas and deep seas across the world. They have high commercial value coupled with increasing global production and trade. Sea cucumbers, informally named as bêche-de-mer, or gamat, have long been used for food and folk medicine in the communities of Asia and Middle East. Nutritionally, sea cucumbers have an impressive profile of valuable nutrients such as Vitamin A, Vitamin B1 (thiamine), Vitamin B2 (riboflavin), Vitamin B3 (niacin), and minerals, especially calcium, magnesium, iron and zinc. A number of unique biological and pharmacological activities including anti-angiogenic, anticancer, anticoagulant, anti-hypertension, anti-inflammatory, antimicrobial, antioxidant, antithrombotic, antitumor and wound healing have been ascribed to various species of sea cucumbers. Therapeutic properties and medicinal benefits of sea cucumbers can be linked to the presence of a wide array of bioactives especially triterpene glycosides (saponins), chondroitin sulfates, glycosaminoglycan (GAGs), sulfated polysaccharides, sterols (glycosides and sulfates), phenolics, cerberosides, lectins, peptides, glycoprotein, glycosphingolipids and essential fatty acids. This review is mainly designed to cover the high-value components and bioactives as well as the multiple biological and therapeutic properties of sea cucumbers with regard to exploring their potential uses for functional foods and nutraceuticals.

## 1. Introduction

In recent decades, the concept of functional foods has offered a new and practical approach to achieving optimal health by promoting the use of natural products with physiological benefits thus reducing the risk of various chronic diseases [1,2].

Most of the currently available functional foods and therapeutic agents are derived either directly or indirectly from naturally occurring sources, especially, the terrestrial food plants and marine species [2–4]. Due to the rich oceanic biodiversity, marine organisms are valuable sources of nutritious foods as well as represent novel reservoirs of biologically active components, in particular bioactive peptides, and antimicrobial, anti-inflammatory and anticancer agents [4–6].

Sea cucumbers are one of the marine animals which are important as human food source, particularly in some parts of Asia [7]. They are usually soft-bodied echinoderms comprising a diverse group of flexible, elongated, worm-like organisms, with a leathery skin and gelatinous body, looking like a cucumber. Habitually, they tend to live on the sea floor in deep seas [8].

A multitude of harvestable sea cucumbers species have been exploited with growing global demand due to their food and pharmaceutical uses [9–13]. The dehydrated sea cucumber is commercially sold, especially in Asian markets with main business in China, followed by Korea and Indonesia and then Japan. On the other hand, these are also exported in appreciable quantities to parts of the United States and northern Australia [14,15]. According to Food and Agriculture Organization of the United Nations (FAO) report Beche-de-mer production and *Apostichopus japonicas* (Selenka, 1867) catches by various countries for the period 1992–2001 was estimated to be 12,331 t (metric ton) (dry weight) [16].

Sea cucumbers, commonly known as trepang, beche-de-mer, or gamat, have long been utilized in the food and folk medicine systems of Asia and Middle East communities [17,18]. Sea cucumbers have been well recognized as a tonic and traditional remedy in Chinese and Malaysian literature for their effectiveness against hypertension, asthma, rheumatism, cuts and burns, impotence and constipation [18–23]. Several unique biological and pharmacological activities namely anti-angiogenic [24], anticancer [25], anticoagulant [26,27], anti-hypertension [28], anti-inflammatory [29–31], antimicrobial [32,33], antioxidant [34], antithrombotic [35,36], antitumor [37,38], and wound healing [39] have been ascribed to chemical compounds extracted from different sea cucumber species. These medicinal benefits and health functions of sea cucumbers can be attributed to the presence of appreciable amounts of bioactive compounds, especially the triterpene glycosides (saponins) [40–42], chondroitin sulfates [43], glycosaminoglycan [26,36], sulfated polysaccharides [44], sterols (glycosides and sulfates) [45], phenolics [46], peptides [47], cerberosides [48] and lectins [49–51].

As far as we know, previously no comprehensive review article as such has ever been published covering the detailed nutritional, medicinal and pharmacological aspects of sea cucumbers. This review is an attempt to mainly compile an inclusive report covering the description of high-value components and bioactives as well as biological and medicinal properties of these multipurpose marine invertebrates, as one of the potential sources for functional foods and nutraceuticals. An updated overview of the distribution, fishery and trade of sea cucumbers is also presented, worldwide.

## 2. Classification, Distribution and Trade

Sea cucumbers belong to the phylum *Echinodermata*, meaning that, they are spiny-skinned, under the class Holothuridea [7,52,53]. The name holothuroid was given by the Greek philosopher, Aristotle (“holos: whole” and “thurios: rushing”). The scientific name “*Cucumis marimus*” which means “sea cucumber” was coined by Pliny (an invertebrate taxonomist) [53]. It was further divided into three subclasses namely Dendrochirotacea, Aspidochirotacea, and Apodacea. There are six orders under these subclasses, named as Aspidochirotida, Apodida, Dactylochirotida, Dendrochirotida, Elasipodida and Molpadiida [15,53,54].

Looking at the oral tentacles is the most common way to separate the subclasses of sea cucumbers. For example, sea cucumbers of subclass Dendrochirotacea have 8–30 oral tentacles while those belonging to Aspidochirotacea may have 10–30 shield like or leaf-like oral tentacles. On the other side, members of Apodacea may contain up to 25 pinnate or simple oral tentacles [15]. As far as the anatomy and distribution is concerned, the length of sea cucumbers is normally 10–30 cm; however some small species of just 3 mm length, and the largest reaching around 1 m, have also been recorded. They are soft and cylindrical-bodied echinoderms which preferably live as dense populations on the deep sea floor and use their tentacles for feeding purposes [8,15,55].

Sea cucumbers are an important component of the marine ecosystem. They are distributed in all oceans the world over, generally living near corals, rocks or sea weeds in warm shallow waters [53,56]. Most of the harvestable species of sea cucumbers, which are mainly targeted as beche-de-mer, belong to two families and seven genera of the Aspidochirotids including *Bohadschia*, *Holothuria* (Holothuridae), *Actinopyga*, *Isostichopus*, *Stichopus*, *Parastichopus* and *Thelenota* (Stichopodidae) and one family and genus of the Dendrochirotids: *Cucumaria* (Cucumariidae) [12].

The total number of presently existing sea cucumber species is about 1250; however, recently, some new species have also been studied from the Indo-Pacific Ocean, being popular as a center for rich biodiversity of Holothuroidea. Besides, there are several undescribed larger sea cucumber species living in shallow water which have not yet been systematically identified because there are rather a small number of holothurian taxonomists [12]. The common and scientific names of some important species of sea cucumbers along with their distribution are given in Table 1.

### 2.1. Sea Cucumber Fishery

By the late 1980s, sea cucumber fisheries had rapidly grown and expanded due to the growing beche-de-mer-related international market, supported by continuing demand of these organisms for aquaculture and biomedical research programs. For example, world over sea cucumber market trends reveal a considerable increase in the trade of sea cucumbers, both in terms of number of species and production yield. In addition to its large expansion in the traditional regions, holothurian fisheries have expanded to a considerable magnitude even in non-traditional fishing areas such as the Galapagos, Mexico and the North America [3–7].

An important and a common feature of subtropical and tropical fisheries, is to target multi-species in shallow water (up to 50 m depth) environments. On the other hand, most temperate fisheries are characterized by single species sea cucumbers, especially, belonging to *Holothuridae* and *Stichopodidae* families which comprise an imperative part of a multi-species invertebrate fishery prevailing in the Indo-Pacific region as long as 1000 years for customary and traditional subsistence uses [12].

The species, having remarkable commercial value, for example, *Holothuria nobilis* (black teatfish), *Holothuria fuscogilva* (white teatfish) and *Holothuria scabra* (sandfish) are mostly distributed in the tropical waters of the Western Pacific and Indian Oceans. The species with average market value include *Actinopyga miliaris* (blackfish), *Actinopyga echinites* (brownfish) and *Thelenota ananas* (prickly redfish) while *Holothuria fuscopunctata*, *Holothuria atra*, *Stichopus chloronotus* and *Stichopus variegates* are some of those with little commercial value. A small, however growing and emergent fishery, for *Isostichopus fuscus*, is recorded in the eastern Pacific regions of Galapagos and Ecuador. Temperate fisheries are divided into, eastern Pacific coasts of North America for *Parastichopus californicus* and *P. parvimensis* (Alaska, Oregon, California and Washington, USA, and British Columbia, Canada), western Pacific regions for *Stichopus japonicus* and a small fishery in the Atlantic for *Cucumaria frondosa* (Maine, USA, and Quebec, Canada) [12].

A multitude of sea cucumber species are being harvested across the regions but the number of commercially exploited species varies broadly. According to research, the highest number (52 and 36) of species is exploited in the Asia and Pacific regions, respectively, of course due to the rich marine diversity in these regions. Little information is available on the biological, ecological and population aspects of most of the commercial species, therefore, in several cases, especially in remote areas, these are marketed without an authenticated taxonomic identification. With regard to the marketing statistics of beche-de-mer international trade, quantitative data are available on fisheries catches. According to the updated trade and catch data available, Asia and the Pacific are the two top sea cucumbers producing regions. Based on the conversion factor employed for calculation of the dry/wet weight of sea cucumbers, it is estimated that the combined harvestable catches for the Asia and Pacific regions are in the range of 20,000 to 40,000 t per year [61].

A substantial share of the world catches (*ca.* 9000 t wet weight per year) is contributed by the temperate regions of the Northern Hemisphere (catches being uphold by one species namely *Cucumaria frondosa*). In this context, African and Indian Ocean regions, with sea cucumber catches contribution in the range of 2000–2500 t per year, are less prominent, while sea cucumber catches is very low in Caribbean and Latin American regions (<1000 t per year) [61,62]. Increasing attention has been focused on evaluating trepang trade in relation to the sustainable management of harvestable sea cucumber resources for imparting socio-economic benefits of this industry to the local fishing communities [62,63]. There are several leading works from Madagascar that reveal an increasing growth in development of sea cucumber aquaculture and trepang trade, due to applying appropriate management strategies [64–66]. Sea cucumbers fishing in Indonesia and elsewhere in Asia is an important segment contributing to improve the economic situation of the Small Islands and local coastal fishing communities; therefore the population of sea cucumbers should be managed appropriately for achieving excellence and sustainable economic and ecological benefits [62,63,67,68].

### 2.2. Sea Cucumber Trades

Most of the sea cucumber exports from the Latin America and the Caribbean regions are from Peru (26.1%) followed by Ecuador (25.9%), Chile (14.1%) and Cuba (10.1%). About 14.0% of sea cucumber exports are derived from countries where either this fishery is banned such as Panama and Costa Rica or have no proper record (Colombia) [63]. According to the FAO (FAO, 2007) report, sea cucumbers catches data is available only for Ecuador, Mexico, Chile and Nicaragua with a total contribution of 6035 t (wet weight) for the period from 1988 to 2005 (Table 2) [61,63].

There is lack of official data regarding the major export destinations for most of the sea cucumber landing, however, it is generally assumed that majority of the catches are supplied to fulfill the Asian (east and southeast Asian regions) market beche-de-mer demands. Of the 52 commercially exploited species, most are tropical and sub-tropical species belonging to the families of Holothuridae and Stichopodidae, including the genus *Actinopyg*a, *Bohadschia*, *Stichopus* and *Holothuria* [68].

The highest Holothuroidea capture landings in the 2000s were yielded by Indonesia, followed by the Philippines. On average basis, almost 47 percent of the world’s Holothuroidea landings per annum, producing an average of 2572 t (wet weight) catches per year, were contributed together by the Philippines and Indonesia during 2000 and 2005. Japan as the largest capture fishery producer of the temperate species (*A. japonicus*), produced an average of 8101 t per year between the period 2000 and 2005. The FAO (Food and Agriculture Organization) sea cucumber capture statistics are usually given on wet weights basis, therefore, the data for landings of sea cucumbers in Southeast Asia seems to be underestimated [68]. This prompts the need to verify whether the data recorded were truly dried and not the wet weight basis.

With the exception of China, where a major portion of sea cucumber production is from aquaculture (*ca*. 10,000 t dry weight/annum), in other Asian countries, the production is derived mainly, if not solely, from capture fisheries [68]. One of the main problems contributing towards the depletion of sea cucumber resources is overfishing. Excluding Japan, other countries of Asia are usually deficient in management practices to conserve and sustain their sea cucumber fisheries. Most importantly, the two major producing countries, the Philippines and Indonesia are lacking specific management measures required for conversation of sea cucumbers. Besides, lack of accurate statistics, habitat loss, global warming and excessive and uncontrolled uses are some of the other currently prevailing threats to sustaining the sea cucumber fisheries resources [68].

Globally, sea cucumber trade specifically intended for the food market has been mostly controlled by China Hong Kong SAR (Special Administrative Region), Singapore and Taiwan Province of China. China Hong Kong SAR have the largest entrepot controlling with contribution of 80 percent of the global import-export sea cucumber trade which might be attributed to the ability of the regions to serve as a corridor for goods to the hinterland of mainland China [68,69]. Traditionally, the lower value products have often been shipped to China Hong Kong SAR for their re-export to China [11,68].

The countries engaged in the trade of sea cucumbers, world over, mostly export trepang to one of the given three main hubs, from where they are re-exported mostly to Chinese consumers worldwide. Almost 90% of the total imports of trepang are contributed by China Hong Kong SAR, China, Singapore, Malaysia, Taiwan Province of China, Republic of Korea and Japan. According to estimates, approximately 80% of the overall international trade is intended primarily for China, Hong Kong SAR [12,68,69]. For example, for the period between 1996 and 2000, 87% of re-exports of trepang from China Hong Kong SAR were destined for China [69].

According to Bruckner *et al.* [12], in 2000 and 2001, 28 countries exported sea cucumbers to Taiwan Province of China while about 50 percent imports to Singapore were from China Hong Kong SAR, with Tanzania, Papua New Guinea and Madagascar being the other main suppliers. Two-way trade is also in practice among the three main exporting centers. China Hong Kong SAR imports highest in1991 (7885 t) were minimum in 1999 (2922 t). For the period between 2000 and 2005, average imports were at around 4626 t. On the other hand, consumption of sea cucumbers in China Hong Kong SAR, peaked in 1991 (4456 t), was decreased from an average value of 3812 t between 1991 and 1994 to a level of 999.9 t between 1995 and 2005.

The species/type, the dried animal size, the extent of dryness (of the processed products) as well as the marketing period of the year, are the important factors in determining the price of sea cucumbers products. For instance, usually prices are 20 to 30% higher just before the Chinese Lunar New Year [69]. It is widely accepted that superior quality products are those from Japan, South Africa, the Pacific coast of South America and Australia while those derived from the Philippines, Indonesia, and China are of lower quality owing to the composition of species and the substandard processing techniques employed [68,69].

According to the FAO global statistics report on sea cucumber, Indonesia is the top most trepang exporter worldwide. About 40–80 percent of the trepangs are exported to China, Hong Kong SAR, with other markets being Japan, Republic of Korea, Taiwan Province of China, Singapore, Malaysia and Australia [67,68]. The average annual prices of Indonesian trepang exported from South Sulawesi during 1996 to 2002 were between USD 15.06/kg to USD 144/kg [67], however, these prices varied (and still vary) greatly depending upon the species and the product specifications. The data from INFOFISH Trade News regarding price trends show that among the high-valued species, sandfish is at the top. INFOFISH Trade News describes only the price for one of the temperate species namely *A. japonicus* with rates almost double than the grade sandfish. The retail price of *A. japonicus* has observed a dramatic increase over the time, for example, the retail price for one kilogram (kg) in 1960, amounting Renminbi (RMB) 18, was as high as RMB 500/kg in 1980 and RMB 3,000/kg (approximately USD 400) in 2004 [70].

Besides, the main trade for the food purposes, there are perhaps hundreds of thousands of sea cucumbers that are marketed for aquarium industry; however information on species, their exact quantities and source countries are rarely available [12].

## 3. Food Value and Important Nutrients

Although there are many cultured and harvestable sea cucumber species, but around 20 species are reported with relatively high economic and food value. Sea cucumbers, usually processed into a dried product know as “bêche-de-mer”, are valued as an important seafood, particularly in Asian countries. Commercially, the product “bêche-de-mer” can be graded into low, medium or high economic value depending upon several aspects such as species, appearance, abudance, color, odor, thickness of the body wall, and market trends and demands [23]. They are widely consumed by people in China, Japan and South Asia [70]. As a food commodity and medicinal cure, sea cucumbers are famous as bêche-de-mer or trepang over many centuries. They are valued as a nutritious dish among the aboriginal people of South East Aisa [7,20]. From nutritional view-point, sea cucumbers are ideal tonic and have an impressive profile of high-value nutrients such as Vitamin A, Vitamin B1 (thiamine), Vitamin B2 (riboflavin), Vitamin B3 (niacin), and minerals, especially calcium, magnesium, iron and zinc [22,53].

Proximate composition of fresh sea cucumbers may differ to a wider extent depending upon the species, seasonal variations and feeding regimes. Typical data as reported in the literature reveal the contents of moisture, protein, fat, ash, and carbohydrates for fresh sea cucumbers to vary from 82.0 to 92.6, 2.5 to 13.8, 0.1 to 0.9, 1.5 to 4.3 and 0.2 to 2.0%, respectively [9]. Commercially processed (dried) sea cucumbers are rich source of crude protein in comparison to most of the seafoods so far in use. Wen *et al.* [23] investigated the chemical and nutritional composition of eight common commercially processed sea cucumber species and found the protein contents to be within the range of 40.7 to 63.3%. The tested sea cucumbers in the given study [23], except *Thelenota anax* and *Actinopyga caerulea* have very low levels of fat (0.3–1.9%) while the ash content is notably high (15.4–39.6%). According to Chen [22], the fully dried sea cucumber material may contain protein content as high as 83% and is sold as nutraceutical in tabulated or capsulated forms [22].

Sea cucumbers contain an interesting combination of valuable amino acids; glycin being the major component (*ca.* 5.57–12.5 g/100 g wet weight) in almost all species identified. Glutamic acid (4.69–7.31 g/100 g wet weight), aspartic acid (3.48–5.06 g/100 g wet weight), alanine (2.95–5.77 g/100 g wet weight) and arginine (2.71–4.95 g/100 g wet weight) are prominent among others [23]. Another important feature of sea cucumber’s amino acids composition is its low lysine/arginine ratio together with high score of essential amino acids (EAAs) due to presence of considerable amount of threonine, tyrosine and phenylalanine [23]. Hypocholesterolemic effects of low lysine/arginine ratio of a protein are well documented. The total amino acids (TAAs) content (33.32–54.13 g/100 g wet weight) [23] as against fatty acids profile, is not so much varied among species, but both of these nutrients as well as polysaccharides and glycosides are higher in intestine and respiratory parts than the body walls. Interestingly, the EAAs/TAAs, EAAs/non-essential amino acids ratios of the intestine and respiratory apparatus are closer to ideal pattern of FAO/WHO [71] suggesting a high nutritive value of sea cucumbers.

## 4. High-Value Bioactives and Therapeutics

During the past three to four decades many efforts have been devoted to isolating numerous biologically active novel compounds from marine sources. Many of such naturally occurring compounds are of great interest for potential drug development as well as an ingredient of new leads and commercially successful products for various industrial applications, especially, pharmaceuticals, agrochemicals, functional foods and nutraceuticals [4]. Sea cucumbers are one of the potential marine animals with high food and medicinal value. The medicinal properties of these animals are ascribed to the presence of functional components with promising multiple biological activities.

A high amount of good-quality protein in sea cucumber is linked with its beneficial effects on serum triglyceride levels [72]. Sea cucumber protein, especially produced from body wall, is rich in glycine, glutamic acid and arginine. Glycine can stimulate production and release of IL-2 and B cell antibody and thus contributes to enhancing phagocytosis. Glycine and glutamic acid are essential components for cells to synthesize glutathione which can stimulate activation and proliferation of NK cell. Arginine can enhance cell immunity by promoting activation and proliferation of T-cell. Due to these amino acid components, sea cucumbers have remarkable function in immune regulation [73]. A major proportion (*ca*. 70 percent) of sea cucumber body wall protein is comprised of collagen. Collagen is recognized as a valued component in the connective tissues, due to its usefulness and specific distribution [47,74]. It can be further converted into gelatin by boiling, to act as a functional bioactive substance [75].

Considerable amounts of phenolics and free radical scavengers have also been determined in sea cucumbers [34,46]. Athunibat *et al.* [34] investigated that aqueous extract derived from sea cucumbers (*Holothuria leucospilota*, *Holothuria scabra*, *Stichopus chlorontus*) contain significantly higher amounts of total phenolics (4.85–9.70 mg gallic acid equivalent (GAE)/g dw) than the organic extracts (1.53–2.90 mg GAE/g dw). Similarly, in another study by Mamelona *et al.* [46], total pheols and flavonoids contents in different parts including digestive tract, gonads, muscles, and respiratory apparatus of sea cucumber, *Cucumaria frondosa*, varied from 22.5 to 236.0 mg GEA/g dw, and 2.9 to 59.8 mg rutin equivalent/g dw, respectively. Acetonitrile-rich fractions and ethyl acetate extracts from digestive tract and water-rich fractions and water extracts from muscles and respiratory apparatus showed the highest amount of total phenols. Among extracts and fractions, acetonitrile-rich fractions exhibited the highest content of phenols for all the tested tissues. As far as the total flavonoids is concerned, the water-rich and acetonitrile-rich fractions from gonads, while water extracts from digestive track and ethyl acetate extracts from muscle and digestive tract have highest levels, among others.

There are a series of other bioactive and antiagent substances in sea cucumbers, such as triterpene glycosides, enzymes, amyloses, fatty acids, cytotoxins, *etc.* with potential capabilities to increase immunity, resist tumor and cruor, protect nerve tissue, ease pain and resist epiphyte as well as contribute to immunopotentiation, anticancer and anticoagulation [71,76].

According to Fredalina *et al.* [77], fatty acids of sea cucumber lipids fractions, are the key components, liable for tissue repair and wound healing properties of this marine animal. Fatty acids profile in terms of myristic (C14:0), palmitic (C16:0), stearic (C18:0), linoleic (C18:2), arachidic (C20:0), eicosapentaenoic (C20:5, EPA) and docosahexaenoic acid (C22:6, DHA) of the lipid fractions, extracted from sea cucumber (*Stichopus chloronotus*) using methanol, ethanol, phosphate buffer saline (PBS), and distilled water, varied largely in relation to the four extraction systems. The PBS extracts showed relatively higher levels of EPA (25.69%) compared to 7.84% in distilled water, 18.89% in ethanol, and only 5.83% in methanol extracts. Amazingly, DHA is present in larger amount (57.55%) in water extracts, as against only 1.20–3.63% in others while it is not detected in ethanol extracts. Interestingly, C16:0 is effectively extracted in ethanol (20.18%) and methanol (20.82%), while 12.55% in PBS and only 2.20% in water extracts. Subsequently, oleic acid (C18:1) is only established in PBS (21.98%) and water extracts (7.50%) [77].

Interestingly, in contrast to vegetable oils which mostly have fatty acids with even carbon numbers, a considerable amount of fatty acids with odd carbon numbers such as C15:0, C17:0, C19:0, C21:0 and C23:1 is also detected in sea cucumber fatty acid profiles [23]. Typically, palmitic acid (C16:0), eicosenoic acid (C20:1 n-9) and arachidonic acid (C20:4 n*-*6) are the dominant components among saturated fatty acids (SFA), monounsaturated fatty acid (MUFA) and polyunsaturated fatty acids (PUFA), respectively in almost all species of sea cucumbers identified [23]. The amount of SFA, MUFA and PUFA among various species is reported to be varied widely. For example, in a study [23] conducted on eight common species of sea cucumbers namely *Stichopus herrmanni*, *Thelenota ananas*, *Thelenota anax*, *Holothuria fuscogilva*, *Holothuria fuscopunctata*, *Actinopyga mauritiana*, *Actinopyga mauritiana*, *Actinopyga caerulea* and *Bohadschia argus*, the contents of SFA, MUFA anad PUFA varied from 31.23 to 61.60%, 27.00 to 45.64% and 5.10 to 23.13%, respectively. The species of sea cucumbers studied in the given work [23], in contrast to those from abyssal, tropical and temperate regions [78,79] have shown higher amounts of SFA and MUFA but lower contents of PUFA. Variations of fatty acid components among species of different sea cucumbers as well as from different regions is understandable and might be linked to factors such as diet, natural habitat climatic conditions and post harvest processing regimes, especially the drying temperature [72]. For commercial purposes, once the sea cucumbers are caught, they usually are gutted, boiled and then dried [12].

Among PUFA of sea cucumbers, arachidonic acid (AA, C20:4 n-6) is detected to be the principal component in almost all species with relatively higher amounts reported for tropical species [78,79]. Medicinal benefits of AA as precursor of eicosanoids and major component of cell membrane phospholipids are well recognized. It is known to play a potential role in growth, and blood clotting process leading to wound healing [80,81]. This supports the sea cucumbers long time utilization as a traditional remedy for burns and cuts in Asia [77]. Presence of considerable amount of eicosapentaenoic acid (EPA) and decosahexaenoic acid (DHA) in several species, especially in tropical and abyssal sea cucumbers [78,89] is medicinally important as these two long chain fatty acids are associated with the reduced incidence of coronary heart diseases and certain cancers[82,83].

Another group of functional substances namely, mucopolysaccharides and chondroitins, have also been identified in sea cucumbers. It has been seen that people suffering from arthritis and connective tissue disorders, are often devoid of these compounds. As such, sea cucumber-derived chondroitin sulfate can be exploited as a nutraceutical to ease joint-pain and arthritis like disorders [84]. It is for this reason that about 3 g/day serving of the dried sea cucumbers is medicinally effective in reducing arthralgia to a significant level [22]. The mechanism of action of chondroitin sulfate is considered to be similar to that of glucosamine sulfate; the latter compound is currently in use as therapeutic agent for easing osteoarthritis [85–87].

Sulfated polysaccharides are reported to exhibit antiviral activity and, based on this fact, Japanese scientists have patented their scientific findings regarding the potential use of sea cucumber chondroitin sulfate to inhibit human immunodeficiency virus (HIV) infection [22,88,89].

Another class of compounds is saponins, commonly identified as holothurins, from sea cucumber. The structural features of these compounds are quite comparable to those of the bioactives from ganoderma, ginseng, and other medicinally popular tonic herbs [22]. They have displayed a wide spectrum of biological effects such as hemolytic, cytostatic, antineoplastic, anticancer and antitumor activities [24,90–93]. Also, one recent study revealed that sea cucumber dietary saponins have shown preventive effect in alleviating the orotic acid-induced fatty liver in rats [94].

Sea cucumbers are rich in glycosides [95], particularly triterpene glycosides which are proven to have antifungal and antitumor activities [96–106]. Sea cucumbers are also known to have impressive amounts of lectins [107,108], cerberosides [109,110], glycosaminoglycans [111–113], sterols and omega-6 sterols and omega-6 and omega-3 fatty acids (EPA and DHA) [45,114,115].

**Figure 1:**
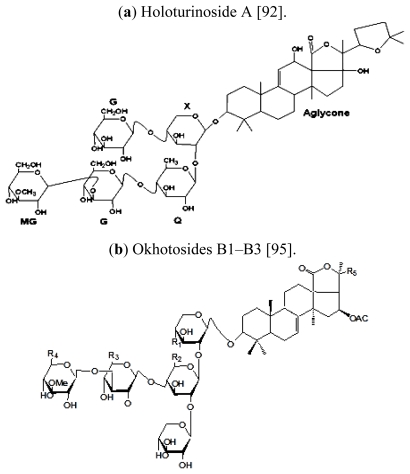

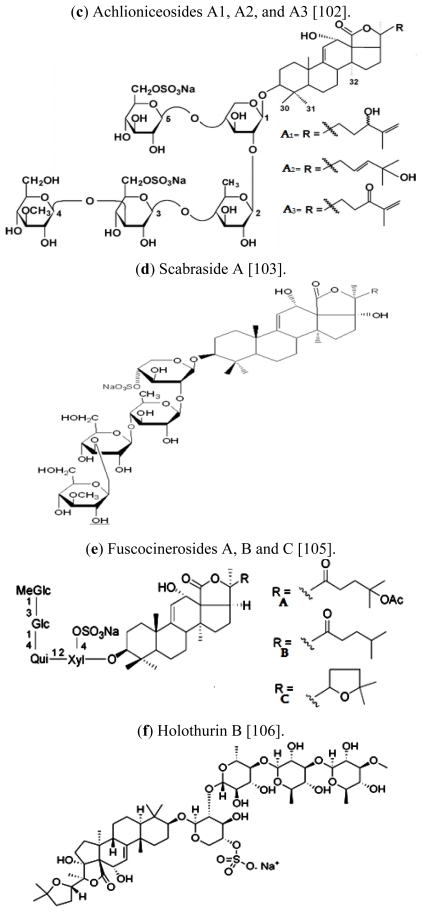

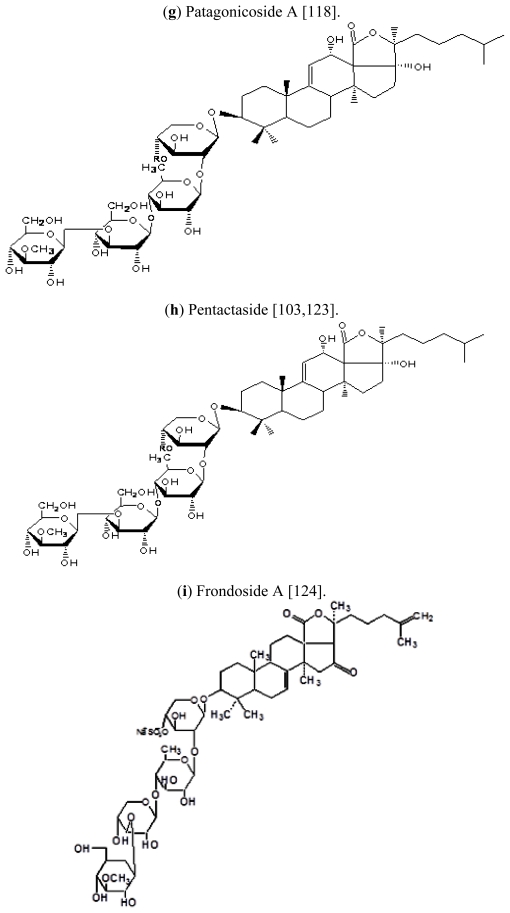

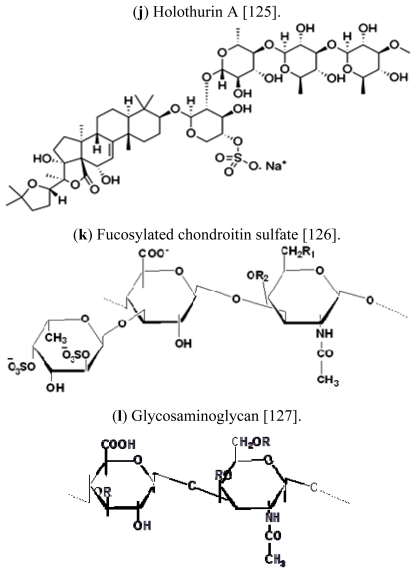
Chemical structures of some of the bioactive compounds identified in sea cucumber.

Recently, a research group, working at Kyushu University, Fukuoka City, Fukuoka, Japan, has discovered three novel compounds (ganglioside molecular species, HLG-1, HLG-2 and HLG-3) in a sea cucumber namely *Holothuria leucospilota*. The newly studied molecules were able to stimulate nerve cell growth in rat cells in the laboratory. The researchers revealed that similar molecules are also present in nine other species of the sea cucumber, as well as the eggs of sea urchins [116]. A number of important bioactive compounds identified in different species of sea cucumber are given in Table 3 while the available chemical structures for some of these are depicted in Figure 1.

## 5. Biological Activities and Medicinal Health Functions

In addition to having a high nutritious value, sea cucumbers have long been recognized in the folk medicine system of Asian countries. An impressive range of medicinal health functions, for example, nourishing the body, tonifying kidney, moistening dryness of the intestines, treatment of stomach ulcers, asthma, hypertension, rheumatism and wound healing have been associated with sea cucumber [22,127–129]. Most importantly, the potential medicinal benefits and multiple biological properties of sea cucumbers are now gaining recognition in modern biomedical research. Scientists believe that sea cucumber extracts are beneficial for human health in different ways and can help reduce the growth of cancer cells [34,130]. In view of the medicinal potential, modern food and pharmaceutical industry is keenly interested to develpe some functional foods and nutraceuticals from different parts of sea cucumbers. Recently, several pharmaceutical firms in Australia initiated the use of sea cucumbers to prevent inflammation [9]. A variety of sea cucumber-derived food and pharmaceutical products are available in South Pacific and Asian markets, including China, Japan, Malaysia and Indonesia [9,77]. In Asia and America dry tablets prepared from the body wall of sea cucumbers are consumed as nutraceuticals for physiological benefit. In Malaysia, boiled skin extracts are consumed as a tonic to treat Asthma, hypertension, rheumatism and wound cuts and burns [9,77]. In addition to health medicinal uses, interestingly, there is much demand for sea cucumber as aphrodisiac food to improve sexual performance [9,77].

Described below are the important biological attributes and medicinal benefits of sea cucumbers as given in the literature.

### 5.1. Anti-Angiogenic

Sea cucumber has emerged as a potential source of anti-angiogenic and anti-tumor agents of medical interest. Recent studies reveal the anticarcinogenic potential of sea cucumber-derived bioactives against certain cancers. Tian *et al.* (2005) [24] examined *in vivo* and *in vitro* anti-angiogenic and anti-tumor functionalities of a newly identified compound philinopside E (PE) from sea cucumber. They assessed through an *in vitro* trial angiogenesis inhibition potential of the compound using different assays such as proliferation, adhesion, migration, tube-formation and apoptosis in PE-treated human umbilical vein endothelial cell (HUVECs) and human microvascular endothelial cells (HMECs). Furthermore, they used *in vivo*, chorioallantoic membran (CAM) assays to examine the PE-inhibition activity on the physiological angiogenesis. Besides, the researchers used western blotting technique to appraise the efficacy of PE on the vascular endothelial growth factor (VEGF) attributing biosignal in HMECs. The results revealed that PE inhibited considerably the proliferation of HMECs and HUVECs, IC_50_ 2.22 ± 0.31 μM and 1.98 ± 0.32 μM, respectively and induced endothelial cell apoptosis at amounts less than 2 μM, showing a concentration-dependent suppression of cell migration and cell adhesion as well as tube formation in HUVECs and HMECs. Similarly, in an *in vivo* CAM assay, PE (5 nM/egg) showed suppression of spontaneous angiogenesis, and exhibited growth inhibition considerably in experimental mouse (sarcoma 180 and hepatoma 22) models. These results show that PE can be explored as an efficient anti-angiogenic agent, for suppressing the active (phosphorylated) forms of vascular endothelial growth factor receptors involved in the endothelial cell survival, adhesion, proliferation and migration.

In another study, the anti-angiogenic activity of a newly isolated sulfated saponin namely Philinospide A, from sea cucumber (*Pentacta quadrangulari*), was tested against angiogensis and tumor growth by Tong *et al.* (2005) [38] in a series of *in vitro* and *in vivo* models. The researchers noted that sea cucumber-derived Philinospide A exhibits anti-angiogenic effects in human microvascular endothelial cells (HMECs) suggesting its uses as a promising anticancer agent. Besides, it possesses dual cytotoxic and antiangiogenic effect, which might be attributed to its inhibitory potential for receptor tyrosine kinases (RTKs). Fucosylated chondroitin sulfate is another compound, identified in the flower and body wall of sea cucumber. This compound offers good antiangiogenic activity, comparable with that of positive control, hydrocortisone/heparin, and even higher than shark cartilage condroitin-6-sulfate [130].

### 5.2. Anticancer/Antiproliferative

Sea cucumbers are reported to contain several compounds with anticancer and antiproliferative properties.

The anticancer activity of three triterpene glycosides, intercedensides A, B, and C isolated from the sea cucumber (*Mensamaria intercedens*) has been evaluated by Zou *et al.* [37]. The isolated triterpene glycosides were structurally elucidated using chemical analyses and NMR and ESIMS spectroscopic approaches. According to the results of the study [37], the given triterpene compounds exhibited cytotoxicity against human tumor cell lines and thus could serve as potential anticancer agents. One of the compounds, intercedenside A also showed good antineoplastic function against mouse S180 sarcoma and mouse Lewis lung cancer.

The hot water extracts from sea cucumber (*Stichopus japonicas*) were tested for their effect on the proliferation and H_2_O_2_ susceptibility of human colon adenocarcinoma Caco-2 cells [131]. The growth of Caco-2 cells was significantly inhibited by the extract treatments. The tested extracts showed concentration-dependent cytotoxity to Caco-2 cells. Cell damage by sea cucumber extract was evident above 1 mg/mL. In addition, coadministration of sea cucumber extracts intensified the H_2_O_2_ cytotoxicity. Another study revealed the isolation of sphingoid bases of sea cucumber (*Stichopus variegatus*) cerberosides along with their cytotoxic effects against human colon cancer cell lines [48]. The isolated cerebrosides were examined for their chemical structure using mass spectroscopic information and found to have a branched C17 to C19 alkyl chain coupled with 1 to 3 double bonds imparting characteristic feature to sea cucumbers sphingoid in comparison to those from mammals. Sea cucumber sphingoid bases showed strong cytotxic activity against cancer cells (DLD-1, WiDr and Caco-2 cells) reducing their viability in a concentration-dependent manner. This activity was comparable to that of sphingosine-treated cells. The tested compounds induced a morphological alteration as condensed chromatin fragments as well as increased the caspase-3 activity, supporting the fact that sphingoid bases can reduce the cell viability by causing apoptosis. It was suggested that sea cucumber-derived bioactive sphingolipids could serve as a functional dietary components to reduce incidence of colon cancer.

Silchenko *et al.* (2007) [95] also studied the anticancer activity of three new triterpene oligoglycosides, okhotosides B1, B2, and B3, isolated from sea cucumber (*Cucumaria okhotensis*), along with the known compounds frondoside A, cucumarioside A2-5, and koreoside A. They used 2-D NMR and MS to elucidate the structures of okhotosides B1-3 on the basis of spectroscopic data established. Their result showed that compounds 1–3 were moderately toxic against HeLa tumor cells, but Frondoside A showed more cytotoxic effect against THP-1 and HeLa tumor cell lines. Similarly, a novel triterpenoid, frondoside A, derived from an Atlantic sea-based sea cucumber species namely *Cucumaria frondosa* has been reported to show an effective growth inhibitory function against human pancreas cancer cells. The proliferation inhibition potential was followed by the magnitude of marked apoptosis. The tested compound (Frondoside A) is supposed to induce apoptosis by means of mitochondrial and cascade activation pathways [132,133].

Althunibat *et al.* (2009) [34] examined the effects of aqueous and organic extracts from three species (*Holothuria leucospilota*, *Holothuria scabra*, *Stichopus chloronotus*) of sea cucumber, on the growth of two human cancer cells: A549 (human non-small lung carcinoma) and C33A (cervical cancer cells) using MTT assay. Of the extracts tested, only *S. chloronotus*-derived extract showed antiproliferative activity against the tested cancer cell lines. Conversely, aqueous extract (AE) from *S. chloronotus* exhibited more toxicity against C33A cells (IC_50_ = 10.0 μg/mL) than A549, whereas AE produced from *H. leucospilota* and *H. scabra* revealed no notable action on the growth of the cancer cells within the concentrations limits employed. On the other hand, sea cucumber extracts produced by the organic solvents inhibited the growth of both the cell lines (A549 and C33A) to varying degrees. The organic extract (OE) from *H. scabra* species offered greater antiproliferative action against A549 and C33A cells with IC_50_ values, 15.5 μg/mL and 3.0 μg/mL, respectively. Furthermore, The OE from *S. chloronotus* showed more cytotoxicity against C33A cells (IC_50_ = 6.0 μg/mL) but little action against A549 cells (IC_50_ = 21.0 μg/mL). Antiproliferative and anticancer functionality of sea cucumber extracts might be ascribed to the presence of considerable amounts of total phenols and flavonids which are valued as effective antioxidants to protect from oxidative stress and degenerative diseases including certain cancers [34].

Janakiram *et al.* (2010) [124] appraised the chemopreventive effects of frondanol A, a glycolipid isolated from sea cucumber (*Cucumaria frondosa*), against azoxymethane-induced rat colon carcinogenesis. They used ACF (aberrantcolonic crypt foci) as an efficacy marker to assess the proliferation expression levels during this study. Besides, the growth-inhibitory and apoptotic effects of frondanol A over concentration range of 10–120 μg/mL using HCT-116 cell line were also studied. Sea cucumber-derived frondanol A exhibited very good growth-inhibitory and apoptotic activities suggesting the uses of this animal as an ingredient for functional foods and nutraceuticals.

Two sulfated triterpene glycosides namely holothurin A (HA) and 24-dehydroechinoside A (DHEA), have been identified in sea cucumber species (*Pearsonothuria graeffei*) by Zhao *et al.* (2010) [134]. Both of these glycosides exhibited sizable influence on metastasis *in vitro* and *in vivo*. Immunocytochemical analyses revealed that both HA and DHEA significantly suppressed the expression of the matrix metallo-proteinase-9 (MMP-9) as well as enhanced the expression level of tissue inhibitor of metalloproteinase-1 (TIMP-1). TIMP-1 is a key regulator for MMP-9 activation. According to the data of Western blot analyses, both HA and DHEA greatly suppressed the expression of VEGF (vascular endothelial growth factor). Both HA and DHEA treatment considerably reduced the adhesion of human hepatocellular liver carcinoma cells (HepG2) to both matrigel and human endothelial cells (ECV-304) and also inhibited HepG2 cell migration and invasion in a concentration-dependent mode. Additionally, HA treatment down regulated the expression level of NF-κB, which might be linked with antimetastatic activity of triterpene glycosides derived from *Pearsonothuria graeffei*.

### 5.3. Anti-Coagulant

Anticoagulant properties of the sea cucumber (*Ludwigothurea grisea*) are linked with the presence of fucosylated chondroitin sulfate in the body wall of this marine animal. This compound has a chondroitin sulfate-like core containing side chains made up of sulfated α-L-fucose attached at Carbon-3 position of the β-D-glucuronic acid [127]. During activated partial thromboplast in time assays (APTT), the given compound showed excellent anticoagulant activity that could be ascribed to its capability to initiate thrombin inhibition by heparin cofactor II and antithrombin. Comparison between the results of chemically modified (desulfated, carboxyl-reduced, and partial defucosylated) and the original polysaccharides indicates that sulfated fucose side branches, play an important role in imparting better anticoagulant properties to fucosylated chondroitin sulfate (FCS). Additionally, strong anticoagulant activity of FCS, coupled with possibly no side effects, makes this polysaccharide a fascinating molecule for potential applications in testing the experimental thrombosis on clinical level.

Mulloy *et al.* (2000) [135] investigated that sea cucumber-isolated fucosylated chondroitin sulfate (FCS), being a kind of sulfated polysacchahers, has potent anticoagulant activity [122]. They used NMR spectroscopy to elucidate the structure of FSC. The result demonstrated that anticoagulant activity of FSC mainly depends on its sulfated fucose branches but a small alteration in determinant sulfate structure can cause loss of almost all anticoagulant action, regardless of the existence of high level of sulfation. In another study [35], the researchers investigated the anticoagulant/antithrombotic action of sea cucumber body wall-derived FCS, and the chemical derivatives of the same polysaccharide, employing a stasis thrombosis model in rabbits. It has been found that both the partial defucosylation and desulfation of the polysaccharides suppressed their anticoagulant action.

Some new fractions have been produced from sea cucumber (*Thelenota ananas*)-derived fucosylated chondroitin sulfate by Wu *et al.* (2010) [136] through a deploymerization process. The newly developed fractions, with varying but narrow molecular weight distribution, were characterized for their physicochemical characteristics using FT-IR and NMR spectral data. The results authenticated the primary structure of the fractions to be retained after the depolymerization. Furthermore, the researchers tested the anticoagulant activity of the fractions produced using the activated partial thromboplast in time and found that APTT activity decreased in a molecular weight dependent manner following a logarithmic-like function. In comparison to high doses of low-molecular weight heparin (LMWH) fraction, a more desirable molecular weight fraction (13,950 Da) displayed lower anticoagulant action. Hence, the fraction showed more efficacy as an antithrombotic agent offering less bleeding risk relative to LMWH.

### 5.4. Anti-Fatigue and Immune Functions

Sea cucumber polypeptides have shown significant anti-fatigue and immune functions in mice; they exhibited no obvious effect on body weight in mice, significantly prolonged the time of loaded-swimming and rolling stick, strongly degraded the content of blood urea nitrogen and increased the content of hepatic glycogen of post exercise mice [137,138]. Liu *et al.* (2009) [139] studied the anti-fatigue and immune functions of sea cucumber oral liquid by determining the loading swimming time and blood lactic acid and hepatic glycogen of mice. Their findings indicated that the swimming time of the mice, administered with oral liquid, in comparison with the control group, was significantly prolonged thus increasing the hepatic glycogen content (*P* < 0.01). Also, after swimming in the test group, a high dose of the oral liquid, the blood lactic acid content of the mice was significantly decreased (*P* < 0.01). The results supported that sea cucumber oral liquid has noticeable anti-fatigue effect.

The bioactives composition of cultured sea cucumber (*Stichopus japonicus*) and its anti-fatigue effect in mice have been elucidated by Bing *et al.* (2010) [140]. The body wall of *S. japonicus* was found to be rich in acidic mucopolysaccharides, collagen, bioactive amino acids and lipids. In comparison with the control group, administration with *Stichopus japonicus*, for 30 consecutive days, prolonged the duration of exhaustive swimming in mice, promoted the synthesis of liver glycogen and hemoglobin and also kept the level of hemoglobin (90 min post-swimming) similar to that of before swimming. It also significantly decreased the generation of blood lactic acid and accelerated the elimination of blood lactic acid and blood urea nitrogen in mice after swimming thus improving the exercise endurance in mice. Based on these findings, it could be concluded that the trepang has an appreciable anti-fatigue activity.

### 5.5. Anti-Hypertension and Angiotensin Converting Enzyme (ACE) Inhibition

Currently, sea cucumbers are gaining recognition among researchers due to their antihypertensive and ACE inhibitory principles. In a recent work, Zhao *et al.* (2007) [75] investigated the antihypertensive effect and purified an ACE inhibitory peptide from sea cucumber (*Acaudina molpadioidea*) gelatin hydrolysate. The gelatin was hydrolyzed sequentially with bromelain and alcalase. The hydrolysate was fractionated into three portions with typical molecular weight ranges (GH-I, <10 kDa; GH-II, <5 kDa; GH-III, <1 kDa) using ultrafiltration membrane bioreactor (UMB). Among the products, the GH-III showed higher ACE inhibitory activity, IC_50_ 0.35 mg/mL. When GH-III was used as drink administered to renal hypertensive rats (RHR) for one month, it significantly reduced the systolic blood pressure and diastolic blood pressure of RHR, indicating anti-hypertensive effect by oral administration. The researchers continued their study [141] further with the aim of preparing hydrolysate of *Acaudina molpadioidea* body wall protein with high anti-hypertensive activity. They hydrolyzed *Acaudina molpadioidea* body wall protein, sequentially, with two enzymes namely bromelain and alcalase and then fractionated the hydrolysate obtained into components with distribution of molecular weight (2 kDa; 2 kDa) using UMBS. The fraction 2 kDa, with superior ACE inhibitory action (IC_50_ of 0.615 mg/mL) was used as drink administered to renal hypertensive rats (RHR) for 30 days. Both the systolic and diastolic blood pressures in RHR were considerably reduced compared with the model group in a dose-dependent manner. Besides, the anti-hypertensive effect, at dosage of 120 μg/g, was as good as for the positive control, captopril (10 μg/g). Overall, it was noted that hydrolysate (GH-III) produced from sea cucumber gelatin has potent ACE inhibitory (*in vitro*) activity and anti-hypertensive (*in vivo*) effects which might have been due to presence of highly bioactive ACE inhibitory peptide.

In another study, Zhao *et al.* (2009) [142], isolated a novel ACE inhibitory peptide from *Acaudina molpadioidea* hydrolysate. The hydrolysate produced was fractionated into two parts with molecular weight range (PH-I, >2 kDa; PH-II, <2 kDa) using an UMB. The PH-II fraction showed higher ACE inhibitory potential. From this PH-II fraction, using various chromatographic techniques (gel filtration, ion-exchange chromatography, RP-HPLC, *etc.*), the researchers isolated an ACE inhibitory peptide. The peptide was further purified and established to be a novel one (sequenced as MEGAQEAQGD), showing negligible resemblance with other ACE inhibitory peptide sequences. After incubation with gastrointestinal proteases, the inhibitory action of the newly characterized peptide was observed to be enhanced by 3.5 times, corresponding to decrease in IC_50_ from 15.9 to 4.5 μM. The tested ACE inhibitory peptide at dosage of 3 μM/kg demonstrated a remarkable anti-hypertensive effect in spontaneously hypertensive rats (SHR).

The antihypertensive and antioxidant activities (*in-vitro*) of two differently processed Icelandic sea cucumber tissues were evaluated and compared by Hamaguchi *et al.* (2010) [28]. The skin, muscle, digestive tract and respiratory tract of sea cucumber (*Cucumaria frondosa*) were processed in different ways yielding aqueous extract and hydrolyzates. The processed sea cucumber products were tested for reducing power, metal ion chelating activity, and ACE activity. According to the results, aqueous extracts, demonstrated higher ACE inhibition compared to the hydrolysates. Different parts of the tested sea cucumber also demonstrated varying magnitude of activities. On the other hand, hydrolysates (process 2) showed higher ORAC (oxygen radical absorbance capacity) values than the aqueous extracts (process 1). They suggested that the higher antioxidative activities of hydrolysates over aqueous extracted samples might be attributed to the presence of antioxidative peptides in addition to other endogenous bioactives in the former case.

### 5.6. Anti-Inflammatory

Studies support that sea cucumber possesses potent anti-inflammatory activity. According to Smith (1978) [143], polian vesicles of sea cucumber (*Holothuria cinerascens*) are known to be the organs attributing inflammatory (including immunologic) receptiveness. As such, they might stand for a rudimentary start of what afterwards progressed into the vertebrate lymphoreticular system. There are also several patents which reveal that tissue fractions of sea cucumber can be exploited as a source of potent therapeutic agents for the treatment of inflammation [29–31]. In an *in vivo* study, Whitehouse and Fairlie (1994) [144] fed the rats of both (male and female) sexes with SeaCare (a human food supplement) composing of dried extracts from selected species of holothurians: 95% w/w sea cucumbers (*Holothuria nobilis*, *Holothuria axiologa* and *Stichopus variegatus*) and 5% w/w sea plant (*Sargassum pallidum*). The anti-inflammatory attributes were tested in rat models of inflammation. Their results indicate that the tested supplement exhibits anti-inflammatory action in both the sexes of rats; however its activity is somewhat lower than the synthetic standard compound (aspirin w/w) against the acute carrageenan-induced paw inflammation. The food supplement was found to be active against adjuvant-induced polyarthritis in rats on a daily dose schedule.

Extracts from sea cucumber species: (*Holothuria tubulosa*, *Leptogorgia ceratophyta*, *Coscinasterias tenuispina* and *Phallusia fumigata*) have been produced using dichloromethane and methanol by Herencia *et al.* (1998) [145] to assess their anti-inflammatory activity. The results showed that the extracts, produced with both the solvents, were effective towards decreasing cyclo-oxygenase activity in inflamed mice tissues but did not modify the constitutive cyclo-oxygenase enzyme. Thus, the tested extracts can be explored as a new marine source for novel anti-inflammatory agents.

### 5.7. Antimicrobial

Sea cucumber extracts have been proven as potential antimicrobial agents in several studies. Antibacterial and antifungal activities of alcoholic extracts of *Actinopyga echinites*, *Actinopyga miliaris*, *Holothuria atra* and *Holothuria scabra* have been studied by Jawahar *et al.* (2002) [121]. The researchers found that except *Bacillus* sp., other strains namely *Escherichia coli*, *Aeromonas hydrophila*, *Enterococcus* sp., *Pseudomonas aeruginosa*, *Klebsiella pneumoniae*, *Staphylococcus aureus*, *Salmonella typhi*, and *Vibrio harveyi*, and fish-generated *Aspergillus* sp. were sensitive to the tested sea cucumber extracts. The antimicrobial potential of these extracts can be ascribed to the presence of antimicrobial agents, most probably, the steroidal sapogenins. Therefore, uses of sea cucumbers, as potential source, for isolation of antimicrobial agents can be suggested. In another study, Ridzwan *et al.* (1995) [146] evaluated the antibacterial activity of the extracts from sea cucumbers harvested from coastal areas of Sabah (Malaysia) using *in vitro* tests. According to their results, both the extracts, the lipid fraction and methanol fraction, derived from sea cucumber species, *Holothuria scabra*, *Holothuria atra* and *Bohadshia argus* did not show considerable antibacterial action. However, PBS (phosphate-buffered saline) derived from *B. argus* and *H. atra*, exhibited significant antimicrobial activity and inhibited the growth of all the tested gram-negative and gram-positive bacteria. The extracts obtained from the outer part of *Holothuria atra*, compared to inner parts, showed weak antimicrobial action.

Antimicrobial activity of the extracts from different body parts of sea cucumber, (*Cucumaria frondosa*), the common starfish (*Asterias rubens*), and green sea urchin (*Strongylocentrotus droebachiensis*) has been examined by Haug *et al.* [147]. The eggs from *Cucumaria frondosa* offered relatively higher antibacterial activity. Several tissues from *A. rubens* exhibited lysozyme-like action, whereas hemolytic activity being observed in almost all the species analyzed. Especially, the body wall has more powerful extracts. A wide variation of bioactivities among the extracts suggests that a variety of substances are capable of antimicrobial functionalities. Therefore, marine echinoderms can be explored as a sustainable natural source for the discovery of novel antibiotic compounds.

In another activity guided research by Kumar *et al.* (2007) [148], methanol extract of sea cucumber (*Actinopyga lecanora*) showed promising antifungal activity, *in vitro*. A new triterpene glycoside, along with two known glycosides, named holothurin B and holothurin A, have been identified in *n*-butanol fractions using repeated column chromatographic fractionation process. Overall, holothurin B showed better *in vitro* antifungal activity against 20 fungal isolates tested including the strain ATCC. Sea cucumber (*Actinopyga lecanora*)*-*based natural products have been recognized to act as a promising source for isolation and identification of antifungal substances. Therefore, sea cucumber-derived holothurin B could be searched as a lead molecule for further development of a potent antifungal drug against infectious diseases. Farouk *et al.* (2007) [149] isolated some bacterial strains from various tissues of the sea cucumber species, (*Holothuria atra*). The bacterial secretions and extracts showed an interesting antibacterial activity. Out of the thirty strains isolated, seven strains exhibited modest to high activity. Researchers also optimized the growth media to enhance the production of antibacterial peptides. Based on activity screening data, the species namely *Klebsiella pneumoniae*, *Salmonella typhimurium*, *Proteus vulgaris* and *Escherichia coli* were found to be the most sensitive organisms.

The crude extracts and pure fractions isolated from *Holothuria polii* (a Mediterranean sea cucumber), have shown concentration-dependent antifungal activity against some molds and yeasts as described by Ismail *et al.* (2008) [150]. According to the data generated, the strains of *Aspergillus fumigatus* were more sensitive to the tested fractions and extracts, whereas those from *Trichophyton rubrum* were less responsive. Besides the extracts, different bioactive compounds, most of them known as triterpene glycosides, have been isolated from sea cucumber offering antimicrobial activity. One of these bioactives, namely patagonicoside A, isolated from sea cucumber (*Psolus patagonicus*) [118], is identified as disulfated tetrasaccharide using 1D and 2D NMR spectral information. Furthermore, it is reported that patagonicoside A has good antifungal activity against pathogenic fungus (*Cladosporium cucumerinum*). Two newly identified sulfated triterpene glycosides, Hemoiedemosides A and B, from the Patagonian sea cucumber (*Hemoiedema spectabilis*) exhibited considerable antifungal activity against phytopathogenic fungus (*Cladosporium cucumerinum*), while the semi-synthetic desulfated derivative hemoiedemosides A was relatively less active [151].

Some secondary metabolites, characterized as triterpene glycosides, from sea cucumber (*Psolus patagonicus*) using a combination of chemical and chromatographic techniques have offered considerable antifungal potential. The purified fractions, mostly comprising of patagonicoside A, showed stronger antifungal action. In comparison with an effective synthetic antifungal product, sea cucumber-derived patagonicoside A and its derivative, for example, desulfated glycoside (ds-patagonicoside A), has comparable antifungal action against molds, *Fusarium oxysporum*, *Cladosporium fulvum*, and *Monilia* sp. [152]. Yuan *et al.* (2009) [153] also reported antifungal activity of four newly identified holostan-type triterpene glycosides, 17α-hydroxy impatienside A, marmoratoside A, marmoratoside B, 25-acetoxy bivittoside D, together with two previously known triterpene glycosides, (impatienside A and bivittoside D), isolated from (*Bohadschia marmorata*) species of sea cucumber. They elucidated the structures of the new triterpene glycosides using spectroscopic data, produced by two-dimensional NMR (2-D NMR) and other biochemical methods. Now the emergence of resistance of bacteria to commonly used synthetic antimicrobial (antibacterial and antifungal) drugs as a result of long-term drug therapy is a common phenomenon. Based upon the antimicrobial potential as revealed by several studies, it would be interesting to explore sea cucumbers as a natural source for isolation of novel antimicrobial agents for drug development against infectious diseases.

### 5.8. Antioxidant

Currently, use of plants or marine-based natural antioxidant compounds has gained much recognition due to their potential health functions and multiple biological properties. Thousands of plants species have already been researched for potential antioxidants; however due to lack of exploration, much potential remains for screening marine organisms for their antioxidant principles [4,6]. Sea cucumber is one of the marine organisms that can be explored as a potential source of valuable antioxidants [34].

The antioxidant potential of fresh and rehydrated sea cucumber (*Cucumaria frondosa*) with/without internal organs has been evaluated by Zhong *et al.* (2007) [154]. The tested sea cucumber exhibited radicals scavenging properties. The rehydrated samples, especially those with internal organs, possessed higher antioxidant activity than their fresh counterparts. According to the findings of this study, poor correlation existed between radical scavenging capacity and total phenolics content, suggesting that other components, in addition to phenolic compounds, could have contributed to the antioxidant activity of sea cucumber. Meanwhile, Zeng *et al.* (2007) [155] reported the antioxidant activity of gelatin hyrolysates from sea cucumber, (*Paracaudina chilensis*). In this study the gelatin was hydrolyzed by bromelain and then using ultrafiltration membrane separated into two major molecular weight fractions (greater than and less than 5 kDa). The hydrolysates tested scavenged the superoxide anion radicals to significant level. A rabbit liver mitochondrial free radical damage model was used for *in vivo* activity trials. Owing to reasonable radical scavenging potential, sea cucumber gelatin hydrolysate prevented the damage of rabbit liver and mitochondria. The antioxidant activity of sea cucumber-derived peptides has been confirmed by Chenghui *et al.* (2007) [156]. They separated sea cucumber hydrolysate into different molecular weight fractions by the methods of ultrafiltration and lyophilization. The results showed that peptides, with molecular weight of 1000~3000 u, exhibited greater antioxidant and scavenging effect on DPPH, even higher than the positive control, Vitamin E.

Total phenolics and total flavonoids contents, and antioxidant activity of the extracts from different parts of Atlantic sea cucumber (*Cucumaria frondosa*) have been assessed by Mamelona *et al.* (2007) [46]. Of the tested extracts, ethyl acetate-extracted components, belonging to digestive tract, showed relatively higher antioxidant activity, while water extracts derived from digestive tract and respiratory apparatus have the least. A good correlation existed between the data of ORAC (oxygen radical absorbance capacity) and total phenolic contents of the extracts/fractions of muscles and gonads. Similarly, ORAC and total flavonoids data showed good correlation (*p* < 0.05) in all experiments. The results of this study showed that *C. frondosa* tissues contained relatively higher levels of natural antioxidants and can be used to prevent lipid oxidation reactions, especially those initiated by free radicals and reactive oxygen species. Hence, sea cucumbers can be a useful natural source for dietary antioxidants. In another investigation, the antioxidant activity and nutritional composition of protein hydrolysates from Atlantic sea-based freez-dried sea cucumber, *Cucumaria frondosa*, has been demonstrated [157]. The hydrolysates tested contained high level of protein (55%), and essential amino acids (35% of total amino acids) along with an impressive nitrogen solubility index (68%). The hydrolysates also indicated significant antioxidant efficacy in both ORAC (267–421 μmol TE/g) and inhibition of lipid oxidation (54–57%) assays, which might be linked to the presence of antioxidant peptides. Atlantic sea-based species of sea cucumber and green sea urchin byproducts could be used as a source of dietary proteins, with potential antioxidant peptides.

A polypeptide isolated from sea cucumber through ultrafiltration and lyophilization methods exhibited effective antioxidant activity when tested on the hydroxyl and superoxide anion radicals [158]. Similarly, the antiproliferative and *in vitro* antioxidant properties of organic extract (OE) and aqueous extract (AE) from sea cucumbers, *Holothuria leucospilota*, *Holothuria scabra* and *Stichopus chloronotus* have been examined by Althunibat *et al.* (2009) [34]. The results indicate that AE of *H. leucospilota* has the highest amount of total phenolics (9.70 mg GAE/g extract), while the OE of *H. scabra* contained the least (1.53 mg GAE/g extract). Also the AE of *S. chloronotus* scavenged DPPH free radical (IC_50_ = 2.13 mg/mL) more effectively while AE (50 mg/mL) from *H. scabra*, *H. leucospilota* and *S. chloronotus* exhibited superior antioxidant activity (77.46%, 64.03% and 80.58%, respectively) in terms of linoleic acid peroxidation. A wide variation of antioxidant components and activities among the analyzed sea cucumber species, have been recorded. Relatively, AE have shown better antioxidant attributes than the OE, supporting that majority of the sea cucumber antioxidant components might have been hydrophilic in nature. It is understandable that the tested sea cucumber species can be employed as a useful source for isolation of natural antioxidant and anticancer agents. According to research work by Wang *et al.* (2010) [159], a gelatin hydrolysate with molecular weight, 700–1700 Da, prepared from sea cucumber (*Stichopus japonicus*) body wall, scavenged the superoxide and hydroxyl radicals in a concentration-dependent manner. The tested gelatin hydrolysate also showed very good inhibitory effect against melanin synthesis and tyrosinase activity in B16 cells. In a similar study Huihui *et al.* (2010) [160] evaluated the free radical scavenging ability of functional polypeptides of sea cucumber (*Acaudina molpadioides*), produced through optimized enzymatic hydrolysis process. In this study more than 70% of the free radicals were scavenged, IC_50_ value for scavenging hydroxyl and superoxide anion free radicals were 27.8 mg/mL, 49.3 mg/mL, respectively. Peptides with molecular weight distribution less than 5 kDa exhibited greater ability to scavenge the free radicals. Besides, there are also other studies which demonstrate that the coelomic fluid from sea cucumber is a good source of antioxidants [161,162].

### 5.9. Anti-Thrombotic

A unique sulfated polysaccharide, extracted from sea cucumber (*Leptopentacta grisea*) body wall has been found to be a strong inhibitor of both P- and L-selectins [126]. This study also supports the findings of a previous work by Zancan and Mourao (2004) [163], that the sulfated fucose branches are required for the anticoagulant and antithrombotic activities of fucosylated chondroitin sulfate (FucCS). The antithrombotic and anticoagulant activities of depolymerized fragment (DHG) of glycosaminoglycan extracted from sea cucumber (*Stichopus japonicas*) (FGAG) have been compared with those of unfractionated heparin (UFH) or low molecular weight heparin (LMWH) by Suzuki *et al.* (1991) [164]. DHG at levels greater than 0.3 mg/kg i.v. significantly prevented the death of mice treated with thrombin (800 U/kg i.v.). Under the same conditions, FGAG, UFH and LMWH prevented the death of mice at dosage higher than 0.3, 0.3 and 0.6 mg/kg i.v., respectively. These results suggest that sea cucumber-derived DHG-1 is a promising antithrombotic agent having quite different anticoagulant property from that of UFH or LMWH.

Another study by Li *et al.* (2000) [165] revealed the antithrombotic effects of sea cucumber-derived glycosaminoglycan (GAG). In this experiment, the effect of GAG on the factors such as assembly, dispersion, and fibrin gel structure and functionality of plasmin was appraised with the aid of electron microscopic and biochemical and chromogenic assays. Besides, the influences of GAG expression and transcription of tissue factor and thrombomodulin in lipopolysaccharide-stimulated human umbilical vein endothelial cells (HUVECs) were also observed. The results of this study reveal that the function of GAG is analogous to dermatan sulfate, both in terms of efficacy and mechanism of antithrombin. Furthermore, it has been shown that coltlysis by GAG is controlled by its capacity to enhance plasmin activity, in order to inhibit the polymerization of fibrin monomer, consequently altering the fibrin network architecture. It can be claimed that such an effect on HUVECs materializes at a transcriptional level and thus might be responsible for the antithrombotic attributes of GAG. The findings of this study suggest that sea cucumber-derived GAG possesses anticoagulant activity *in vivo* and can be used as a promising drug for antithrombotic therapy.

### 5.10. Antitumor

Sea cucumbers contain a variety of anti-tumor ingredients. These anti-tumor active components play important roles in different stages of tumor development, progression and metastasis. The exploration of anti-tumor active ingredients from sea cucumbers might open windows of opportunities to discover new antitumor agents from other marine sources for clinical tumor treatment [166]. Triterpene glycosides, namely holothurinosides A, B, C and D as well as desholothurin A from sea cucumber (*Holothuria forskali*), have considerable antitumor activity [167]. Holothurinosides A and B are the first non-sulfated pentasaccharide saponins isolated from marine echinoderms while C and D are the di and tetrasaccharides. Sea cucumber-derived holothurinosides A–D and the related saponin have shown antitumor and antiviral activities. Holothurinosides A and desholothurin A are the most effective with IC_50_ values of 0.46 and 0.38 mg/mL, respectively against P388 cell lines. Similarly, five new saponins (holothurinosides A–D**)** isolated from the aqueous-methanolic extract of sea cucumber (*Holothuria forskali*) have also offered considerable antitumor and antiviral activities.

According to another research report [120], the glycoproteins obtained from the body wall of sea cucumber (*Mensamaria intercedens*) could significantly inhibit the growth of Sarcoma 180 cells implanted subcutaneously in mice (*p* = 0.05) at dosage of 20–30 mg/kg per day ×10 with no sign of toxicity. Six newly isolated triterpene glycosides, intercedensides D–I, from the whole body of sea cucumber (*Mensamria intercedens*), have shown good antitumor activity [37]. Chemical and spectroscopic (NMR and ESIMS) structural elucidation demonstrated that intercedensides D, E, G, and H have conjugated double bond system (22*Z*,24-diene) in the aglycon side chain, while intercedensides F and I, contained only a single double bond in the same chain. Lntercedensides D–H has displayed considerably high cytotoxicity (ED_50_ 0.96–5.0 mg/mL) against human tumor cell lines. The effect of philinopside A, a novel sulfated saponin derived from sea cucumber (*Pentacta quadrangulari*) on the angiogenesis and tumor growth have been studied by Tong *et al.* (2005) [38] using different *in vitro* and *in vivo* models. The results revealed that philinopside A has high anti-tumor activity in both the *in vivo* and *in vitro* trials.

According to Ogushi *et al.* (2006) [168], when human colon adenocarcinoma Caco-2 cells were exposed to hot water extract of sea cucumber (*Stichopus japonicus*), certain morphological changes occurred in the extract-treated cells. The researchers in this study demonstrated the induction of apoptosis using phosphatidylserine translocation (APO Percentage Assay kit), terminal deoxynucleotide transferase-mediated dUTP-biotin nick-end labeling (TUNEL), and DNA fragmentation as DNA ladder. The data showed that apoptosis is induced by a high molecular weight fraction in a dose dependent manner. It could be predicted that water extracted (water-soluble) and higher molecular weight compounds of sea cucumber might exhibit anti-tumor activity by triggering apoptosis, and the apoptosis-inducing activity may contribute to cancer chemopreventive effects of sea cucumber.

In another experiment conducted by Zhang *et al.* (2006) [105], active *n*-BuOH extract of sea cucumber, (*Holothuria fuscocinerea*) was fractionated resulting in isolation of three new triterpene glycosides, fuscocinerosides A, B, and C, along with two known glycosides, pervicoside C and holothurin A. Structural elucidation, using spectral and chemical data showed that all the compounds possessed the same tetrasaccharide moiety, 3-*O*-methyl-β-D-glucopyranosyl-(1→3)-β-D-glucopyranosyl- (1→4)-β-D-quinovopyranosyl-(1→2)-4-*O*-sodiumsulfato-β-D-xylopyranosyl, linked to C-3 of holostane triterpene aglycones that differed in their side chains and 17-substituents. All the tested glycosides exhibited considerable cytotoxicity *in vitro* against human tumor cell lines [99]. Wu *et al.* (2006) [169], elucidated the structure of three newly isolated triterpene glycosides (nobilisides A, B and C) from sea cucumber, (*Holothuria nobilis*). Various spectral and chemical analyses were performed to deduce the chemical structures of the compounds isolated. Their results revealed that compounds A and C are non-sulfated monoglycosides while B is a sulfated diglycoside. All the three glycosides exhibited notable cytotoxic effects against human tumor cells. In their next study [170], they identified hillasides A and B, as new triterpene glycosides, in sea cucumber (*Holothuria hilla*) along with a previously known glycoside, holothuria B. They found that occurrence of conjugated double bonds [22*E*,24-diene] in the aglycone of hillasides A is a unique structural feature among sea cucumber glycosides. Both of the newly identified glycosides showed appreciable cytotoxic potential against tumor cell lines in human.

Recently, a new cytotoxic lanostane-type triterpene glycoside from the sea cucumber (*Holothuria impatiens*) has been isolated and structurally identified [171]. The newly elucidated compound showed *in vitro* cytotoxicity, even better than that of an anticancer drug etoposide (V-16) against seven human tumor cells. In a recent investigation, Lu *et al.* (2009) [122] evaluated the antitumor activity of *Stichopus japonicus* acid ingredients mucopolysaccharide (SJAMP) involving animal experimental trials. Their results revealed SJAMP to be a potential antitumor agent. SJAMP is one of the important biologically active compounds identified in sea cucumber, (*Stichopus japonicas*). Based on the facts, sea cucumbers can be recommended as a medicated food with therapeutic functions during and after the treatment of certain tumors. Aminin *et al.* (2010) [42] identified a new immunomodulatory lead compound, cumaside from sea cucumber (*Cucumaria japonica*). Chemically, cumaside is a complex of monosulfated triterpene glycosides and reveals antitumor activity against experimental mouse *Ehrlich carcinoma in vivo* [42].

### 5.11. Antiviral

There are evidences that sea cucumbers bioactives also have antiviral activity. The antiviral activity of Liouvillosides A and B, which are trisulfated triterpene glycosides, isolated from Antarctic sea cucumber (*Staurocucumis liouvillei*), have been examined by Maier *et al.* (2001) [117]. Based on the results of activity-directed bioassays, both glycosides showed good antiviral activity against herpes simplex virus type 1 (HSV-1). Sea cucumber-derived fucosylated chondroitin sulfates (FCS), recognized as a type of sulfated polysacchahers [35,135], can inhibit human immunodeficiency virus (HIV) infection, thus suggesting potential utilization of these valuable marine invertebrates as a natural therapy against HIV disorders and AIDS (acquired immune deficiency syndrome) [88,89].

### 5.12. Osteoarthritis

It is revealed that certain chemical compounds namely chondroitin, mucopolysaccharides and glucosamine, occurring in sea cucumbers, have beneficial effects in arthritis disorders. Researchers have shown that usage of sea cucumber is beneficial in maintaining prostaglandins balance thus helping out in the treatment of musculo-skeletal inflammatory disorders such as osteoarthritis, rheumatoid arthritis and spinal arthritis [85–87]. Two types of fucan sulfates have been isolated from sea cucumber (*Stichopus japonicus*) body wall using chloroform/methanol solvent system. Both types of fucan sulfates tested inhibited the osteoclastogenesis in an *in vitro* assay. This suggests that these compounds derived from sea cucumber are strong inhibitors of osteoclastogenesis [85]. Therefore, sea cucumber-derived chondroitin sulfate and other related marine compounds can be a useful folk remedy for curing joint-pain and arthritis. The intake of dried sea cucumber is medicinally effective in suppressing arthralgia [85–87].

### 5.13. Wound Healing

Sea cucumber and sea cucumber-based products are now becoming available in shelves of health food stores due to their therapeutic effects, in particular the wound healing functions (to speed recovery of sores, cuts and wounds on the skin, as well as internally for ulcers and other ailments that involve internal damage). It is believed that direct use of sea cucumber can reduce wound recovery time and help new tissue formation and regeneration in human just as the sea cucumber’s ability to quickly regenerate its own body tissue when damaged [53,172,173]. It is evident that sea cucumber (*Stichopus chloronotus*) fatty acids including arachidonic acid (AA C20:4), eicosapentaenoic acid (EPA C20:5), and docosahexaenoic acid (DHA C22:6) can play a potential role in tissue repair and wound healing [9,77]. It has been revealed in the literature that the bottom sediment feeder sea cucumber can contain high contents of branched chain fatty acids (BCFA) to assist in the potential wound healing activity [9]. An appreciable amount of EPA in sea cucumbers [9,23,77] might be linked well with the ability of these echinoderms to initiate tissue repair. EPA is known to be the main active compound in fish oils, and exerts its function by means of prostaglandin inhibition and anti-thrombic attribute. Besides, EPA also plays a potential role in the mechanism of blood-clotting [77,80,81].

### 5.14. Other Properties

Besides, the pharmacological and therapeutic attributes described above, there are other studies which revealed that sea cucumbers have further potential biological properties such as antihistamine [174], analgesic anti-anaphylactic [174,175], antinocieptive [176,177] and antileishmanial [178]. For example, after the intake of sea cucumber pellet, histamine-induced anaphylactic shock guinea pigs showed a reduction of anaphylaxis [174]. Extracts from selected sea cucumber (*Stichopus* sp.), revealed potential analgesic activity [7,174,175]. Ridzwan *et al.* (2001) [176] and Ridzwan *et al.* (2003) [177] investigated that water extracts from sea cucumbers (*Holothuria leucospilota*, *Bohadschia marmorata* and *Bohadschia vitiensis*) as well as the coelomic fluid from *Stichopus hermanii* have antinociceptive effect in mice. In another study [178], methanol extract and *n*-butanol fraction of sea cucumber (*Actinopyga lecanora*) exhibited excellent Leishmania donovani inhibition activity *in vitro* and *in vivo* suggesting the uses of these multipurpose marine invertebrates as platform for the development of antileishmanial drugs from some other potential marine resources. Some important pharmacological and medicinal properties of sea cucumbers-derived bioactives are presented in Table 4.

## 6. Future Prospectives

Currently, the uses of sea cucumbers due to their potential health benefits to humans, are gaining much recognition among consumers, medical and biomedical researchers. The south Asian region communities, especially the older generation, consume sea cucumber and its coelomic fluid, even though some of them are not aware of the medicinal effects associated with it. In peninsular Malaysia, the medicinal uses of sea cucumbers (mainly *Stichopus hermanni* and *S. horrens*), locally known as gamat (Stichopidae family), have been exploited; however such applications needs to be proven on a scientific basis using some clinical models.

Although some preliminary data on nutritional attributes and bioactives of sea cucumber is available, this marine animal, due to its species diversity, availability and medicinal utility, remains a highly potential candidate for the search of novel marine compounds for drug discovery. So far, numerous studies have been conducted on sea cucumber, but still potential exists to isolate and identify new compounds from different parts of various species of this harvestable marine invertebrate. For example, there is a need to fully identify and characterize the profile of sea cucumber’s alkaloids, antioxidant phenolics, functional peptides and other health-promoting components for their chemical structure and detailed biological properties using state-of-the-art spectroscopic and biomedical approaches and bioactivity-directed assays.

Usage of by-products in food to improve their health promoting properties is a potential field. In this case, isolation and production of compounds from sea cucumber, in high class purity, could lead to develop functional foods. It has been proven that by-products from echinoderms as well as sea cucumbers contain considerable amounts of different nutritiously important components. Therefore, efforts should be devoted to explore the potential uses of sea cucumber-based biological wastes for value-addition. The magnitude/volume of sea cucumbers currently in use for medicinal, pharmaceutical, cosmoceutical and nutraceutical purposes is still not reflected in the literature and needs to be documented.

There are several research groups engaged in conducting preliminary studies on anti-angiogenic, anticoagulant, anticancer, ACE inhibitory, anti-inflammatory and antitumor, *etc.,* activities of the sea cucumber. It is important to identify, isolate and elucidate the structure of related bioactives and the mechanisms involved for all such medicinal effects using more spectrochemical evidences and activity directed protocols as well as clinical human models. The antinutritional factors, if any, related to the underutilized or unexplored sea cucumber species should be appraised. There is also prompt need for authentication of nomenclature of many such underutilized or newer sea cucumber species. Most importantly, sea cucumbers dietary intake and nutraceutical/medicinal dosages should be standardized on human clinical basis for attaining optimum functionality and physiological benefits.

## 7. Conclusions

Sea cucumbers are marine invertebrates that have gained popularity among researchers in recent decades, not only due to their nutritive value, but also due to their potential health benefits and therapeutic uses. Extensive literature survey revealed that sea cucumber has a long history as a traditional food and folk medicine. Most of the sea cucumber uses have been validated through scientific and ethno-pharmacological research. A myriad of bioactives, isolated from sea cucumber such as chondroitin sulfate, triterpene glycosides (saponins), lectins, heparin, cerberosides**,** ganglosides**,** bioactive peptides, sterols and omega-6 and omega-3 fatty acids, have shown multiple biological activities such as anti-angiogenic, anticancer, anticoagulant, anti-hypertension, anti-inflammatory, antimicrobial, antifungal, antioxidant, antithrombotic and antitumor. Overall, we concluded that sea cucumber can be explored as a potential source of high-value components for functional foods, and the nutraceutical industry. There is a great potential to utilize sea cucumbers to develop valuable functional foods with physiological benefits for human beings. The current review emphasizes that this underutilized resource contains a wide array of functional bioactives that can be isolated and purified to act as ingredients of functional foods and nutraceuticals.

## Figures and Tables

**Table 1:** The common and scientific names and distribution of common sea cucumber species.

Scientific (Binomial) name	Common name	Distribution	References
*Actinopyga echinites* (Jaeger, 1833)	Brown fish (deep water red fish)	South-Pacific	[12]
*Actinopyga lecanora* (Jaeger, 1833)	Stone fish	Indo-West Pacific and South China Sea	[12,57]
*Actinopyga mauritiana* (Quoy & Gaimard, 1834)	Surf red fish, White-spotted or Speckled sea cucumber	Indo-Pacific, South China Sea, Africa and Hawaii	[12,58]
*Actinopyga miliaris* (Quoy & Gaimard, 1833)	Black fish	South-Pacific	[12]
*Actinopyga obese* (Selenka, 1867)	Plump sea cucumber	Western and Central Pacific and Hawaii	[58]
*Bohadshia argus* (Jaeger, 1833)	Spotted or Argus fish, Leopard sea cucumber	Indo-Pacific South-Pacific, South East and South China Sea	[12,57,58]
*Bohadshia graeffei* (Semper, 1868)	Orange fish	South-Pacific, South East Asia	[12]
*Bohadshia marmorata* (Jaeger, 1833)	Chalky fish/chalky sea cucumber	Indo-Pacific, South-Pacific, Red Sea and South China Sea	[12,57]
*Bohadschia paradoxa* (Selenka, 1867)	Paradoxical sea cucumber	Indo-Pacific and Hawaii	[58]
*Bohadshia vitiensis* (Semper, 1868)	Brown sandfish	South-Pacific, Indian Ocean	[12]
*Cucumaria frondosa* (Gunnerus, 1767)	Phenix sea cucumber, pumpkins; orange footed sea cucumber	Indo-West Pacific, and North East coast of Scotland, Shetland and Orkney, West Atlantic	[12,57,59]
*Holothuria arenicola* (Semper, 1868)	Sand sea cucumber	Indo-Pacific and Tropical West Atlantic	[58]
*Holothuria atra* (Jaeger, 1833)	Lollyfish or Black sea cucumber	Indo-Pacific, South-Pacific, South China Sea, Persian Gulf, Africa, Red Sea to Hawaii	[12,58]
*Holothuria cinerascens* (Brandt, 1835)	Ashy sea cucumber	Indo-Pacific, South China Sea, Red sea to Hawaii	[58]
*Holothuria dificillis* (Semper, 1868)	Difficult sea cucumber	Indo-Pacific, South China Sea, Red Sea to Hawaii	[58]
*Holothuria edulis* (Lesson, 1830)	Burnt hotdog or Pink fish	Indo-Pacific, South-Pacific, South China Sea, Red Sea to Hawaii	[12,58]
*Holothuria fuscogilva* (Cherbonnier, 1980)	White teatfish	South-Pacific, Indian Ocean, South East Asia	[12]
*Holothuria fuscopunctata* (Jaeger, 1833)	Elephant trunkfish	South-Pacific, South East Asia	[12]
*Holothuria hilla* (Lesson, 1830)	Light-spotted sea cucumber	Red Sea to Hawaii, Indo-Pacific, South-Pacific, South China Sea, Persian Gulf	[58]
*Holothuria impatiens* (Forskaal, 1775)	Slender sea cucumber or Impatient sea cucumber	Indo-Pacific, Persian Gulf, South China Sea, Southern California, Hawaii, Caribbean (Mexico) and other Tropical Waters	[12,58]
*Holothuria Mexicana* (Ludwig, 1875)	Donkey dung	Caribbean (Venezuela)	[12]
*Holothuria nobilis* (Selenka, 1867)	Black teatfish	Indo-Pacific, South Pacific, South China Sea, SE Asia, Red Sea to Hawaii, Africa	[58]
*Holothuria pardalis* (Selenka, 1867)	Leopard sea cucumber	Indo-Pacific and Eastern Pacific, Red Sea to Hawaii	[58]
*Holothuria pervicax* (Selenka, 1867)	Stubborn sea cucumber	Indo-Pacific, Africa and Hawaii	[58]
*Holothuria scarab* (Jaeger, 1833)	Sandfish	Africa, Red Sea, South China Sea, South-Pacific, South East Asia, Indian Ocean	[12,58]
*Holothuria scabra versicolor* (Conand, 1986)	Golden sandfish	South-Pacific, South East Asia	[12]
*Isostichopus badionotus* (Selenka, 1867)	Three-rowed sea cucumber	Caribbean (Venezuela)	[12]
*Stichopus californicus* (Stimpson, 1857)	Giant red sea cucumber	East Pacific (US/Canada)	[12]
*Stichopus chloronotus* (Brandt, 1835)	Black knobby or green fish	Indo-West Pacific, Eastern Africa to Hawaii (rarely), Indian Ocean and the South-Pacific	[12,58]
*Stichopus hermanni* (Semper, 1868)	Curry fish or Hermann’s sea cucumber	Indo-West Pacific, South East Asia and South-Pacific	[12,58]
*Stichopus japonicus* (Selenka, 1867)	Japanese sea cucumber	North West pacific and Japan Coastal Areas	[12,60]
*Stichopus horrens* (Selenka, 1867)	Golden sea cucumber	Indo-Pacific, South-Pacific and Hawaii	[58]
*Stichopus mollis* (Hutton, 1872)	New Zealand sea cucumber	New Zealand, Australia, Tasmania	[12]
*Thelenota ananas* (Jaeger, 1833)	Prickly redfish	South-Pacific	[12]
*Thelenota anax* (Clark, 1921)	Amber fish	South-Pacific	[12]

**Table 2:** Volume of sea cucumbers catches (in tons) reported to FAO by Chile, Ecuador, Mexico and Nicaragua in comparison to the total tonnage reported worldwide.

Year	Nicaragua	Ecuador	Mexico	Chile	Total in the Region	World harvest total	Percentage from region
1988	-	3	-	-	3	19,905	0.02
1989	-	10	-	-	10	17,467	0.05
1990	-	12	-	-	12	19,976	0.06
1991	-	29	-	-	29	21,790	0.15
1992	-	152	-	237	389	20,892	1.95
1993	-	12	-	13	25	19,348	0.13
1994	-	12	-	4	16	24,505	0.08
1995	-	12	-	106	118	24,050	0.59
1996	-	12	-	115	127	26,795	0.64
1997	-	15	-	1	16	24,672	0.08
1998	-	15	271	30	316	22,004	1.59
1999	-	15	234	108	357	20,462	1.79
2000	-	15	426	1510	1951	24,509	9.80
2001	-	15	481	107	603	20,431	3.03
2002	-	15	290	106	411	23,445	2.06
2003	-	15	285	307	607	28,085	3.05
2004	-	15	265	234	514	27,540	2.58
2005	51	15	312	153	531	26,002	2.67
Total	51	389	2564	3031	6035	411,878	1.46

*Source*: FAO Fisheries and Aquaculture Information and Statistics Service 2007.

**Table 3:** Medicinally important bioactives in different species of sea cucumbers.

Bioactive compounds	Sea cucumber species	References
Triterpene glycoside (Saponin)	*Pentaca quadrangularis*, *Holothuria atra*, *Actinopyga echinites*, *Bohadschia subrubra*, *Pearsonothuria graeffei* (*Holothuria forskali*), *Psolus patagonicus*, *Mensamria intercedens*, *Thelenota ananas*, *Holothuria fuscocinerea*, *Holothuria nobilis*, *Holothuria hilla*, *Holothuria impatiens*, *Cucumaria frondosa*, *Holothuria leucospilota*	[24,25,37,38,40,41,90–94,96–106]
Sulfated triterpene glycosides	*Hemoiedema spectabilis*, *Cucumaria japonica*, *Staurocucumis liouvillei*	[117,118]
Cerberoside	*Bohadschia argus*	[48,109,110]
(Fucosylated) Chondroitin sulfates	*Ludwigothurea grisea*, *Thelenota ananas*, *Pearsonothuria graeffei*, *Stichopus tremulus*, *Holothuria vagabunda*, *Isostichopus badionotus*	[27,35,36,43,84–87]
Glycosaminoglycan	*Stichopus japonicas*, *Holothuria* (*Metriatyla*) *scabra*, *Thelenota ananas*	[26,36,111–113]
Lectin	*Stichopus japonicus*, *Holothuria atra*, *Holothuria scabra*	[49–51,107,108]
Sulfated polysaccharide	*Ludwigothurea grisea*, *Stichopus japonicus*	[44,88,89,119]
Sterol (glycosides, sulfates)	*Cucumaria frondosa*	[45,114,115]
Bioactive peptides {protein (gelatin & collagen) hydrolysates}	*Cucumaria frondosa*, *Acaudina molpadioides*, *Paracaudina chilensis*, *Acaudina molpadioidea*, *Stichopus japonicas*	[31,32,47,73–75]
Phenols and flavonoids	*Holothuria scabra*, *Holothuria leucospilota*, *Stichopus chloronotus*, *Cucumaria frondosa*	[34,46]
Triterpene oligoglycosides	*Cucumaria okhotensis*	[95]
Glycoprotein	*Mensamaria intercedens*	[120]
Steroidal sapogenins	*Actinopyga echinites*, *Actinopyga miliaris*, *Holothuria atra*, *Holothuria scabra*	[121]
Mucopolysaccharide (SJAMP)	*Stichopus japonicas*	[122]
Polyunsaturated fatty acids (PUFA): arachidonic acid (AA C20:4 n-6), eicosapentaenoic acid (EPA C20:5 n-3), docosahexaenoic acid (DHA C22:6 n-3)	*Stichopus herrmanni*, *Thelenota ananas*, *Thelenota anax*, *Holothuria fuscogilva*, *Holothuria fuscopunctata*, *Actinopyga mauritiana*, *Actinopyga caerulea*, *Bohadschia argus*, *Stichopus chloronotus*, *Holothuria tubulosa*, *Holothuria polii*, *Holothuria mammata*	[9,23,77–83]

**Table 4:** Pharmacological and medicinal activities of bioactive compounds from sea cucumbers.

Sea cucumber species	Bioactive compounds	Pharmacologica/medicinal activity	References
*Pentacta quadrangularius*, *Cucumaria frondosa*	Sulfated saponin [Philinospide A], Philinospide E (PE), Sea cucumber fractions: B1000 and Fucosylated chondroitin sulfate	Antiangiogenic	[24,38,130]
*Holothuria scabra*, *Holothurialeucos pilota*, *Stichopus chloronotus*, *Cucumaria frondosa*, *Cucumaria okhotensis*, *Mensamaria intercedens*, *Pearsonothuria graeffei*, *Stichopus japonicus*, *Stichopus variegates*	Triterpenoid [Frondoside A], Triterpene oligoglycosides [Okhotosides B1, B2, and B3], Triterpene glycosides [Intercedensides A, B, and C], Glycolipid [Frondanol A], Triterpene oligoglycosides [Holothurin A and 24-dehydroechinoside], Frondanol(R)-A5p, sphingoid base composition of cerebrosides	Anticancer	[34,37,48,124,131–134]
*Ludwigothurea grisea*, *Thelenota ananas*	Fucosylated chondroitin sulfate, Fucosylated chondroitin sulfate	Anticoagulant	[127,135,136]
*Stichopus japonicas*	Low molecular weight polypeptides, Polypeptides Acidic mucopolysaccharides, collagen and bioactive amino acids (all together)	Anti-fatigue	[137–140]
*Actinopyga echinites*, *Actinopyga miliari*, *Holothuria atra*, *Holothuria scabra*, *Bohadshia argus*, *Cucumaria frondosa*, *Holothuria poli*, *Hemoiedema spectabilis*, *Psolus patagonicus*, *Actinopyga lecanora*, *Holothuria atra*, *Psolus patagonicus*, *Bohadschia marmorata*, *Cucumaria frondosa*	Steroidal sapogenins, (Phosphate-buffered saline [PBS]), Sulfated triterpene glycosides [Hemoiedemosides A and B], Triterpene glycoside [patagonicoside A], Triterpene glycoside [holothurin B (saponin)], Triterpene glycoside [patagonicoside A], Holostan-type triterpene glycosides [marmoratoside A, 17α-hydroxy impatienside A, impatienside A and bivittoside D], Bioactive peptides	Antimicrobial Antibacterial and antifungal	[32,118,121,146–153]
*Cucumariafrondosa*, *Stichopus japonicus*, *Paracaudina chilensis*, *Cucumaria frondosa*, *Cucumaria frondosa*, *Holothuria scabra*, *Holothuria leucospilota*, *Stichopus chloronotus*, *Acaudina molpadioides*	Gelatin hydrolysate, Gelatin hydrolysate, Protein hydrolysate [bioactive peptides], Bioactive peptides, Phenols and flavonoids, Phenols, Gelatin hydrolysate [Bioactive peptides], Collagen polypeptides	Antioxidation	[34,46,154–160]
*Stichopus japonicas*	Glycosaminoglycan, Holothurian glycosaminoglycan	Antithrombotic	[126,163–165]
*Mensamaria intercedens*, *Mensamaria intercedens*, *Holothuria hilla*, *Pentacta quadrangularis*, *Holothuria forskali*, *Stichopus japonicus*, *Holothuria nobilis*, *Holothuria fuscocinerea*, *Stichopus japonicus*, *Holothuria impatiens*, *Ludwigothurea grisea*, *Cucumaria japonica*	Triterpene glycosides, [intercedensides D–I], Glycoprotein (GPMI I), Triterpene glycosides [hillasides A and B], Sulfated saponins [Philinopside A], Triterpene glycosides [holothurinosides A, B, C and D; and desholothurin A], Mucopolysaccharide (SJAMP), Triterpene glycosides [nobilisides A, B and C], Triterpene glycosides [fuscocinerosides A, B, and C], Monosulfated triterpene glycosides, Lanostane-type triterpene glycoside [impatienside A], Sulfated polysaccharide, Monosulfated triterpene glycosides [cumaside]	Antitumour	[37,38,42,120,122,166–171]
*Staurocucumis liouvillei*	Trisulfated triterpene glycosides [liouvillosides A and B]	Antiviral	[117]
*Stichopus japonicas*	Fucan sulfate, Glucosamin, Chondroitin	Osteoarthritis	[85]
*Thyone briareus*, *Stichopus chloronotus*, *Stichopus herrmanni*, *Thelenota ananas*, *Thelenota anax*, *Holothuria fuscogilva*, *Holothuria fuscopunctata*, *Actinopyga mauritiana*, *Actinopyga caerulea*, *Bohadschia argus*, *Stichopus chloronotus*, *Holothuria tubulosa*, *Holothuria polii*, *Holothuria mammata*	Polyunsaturated fatty acids, (arachidonic acid, eicosapentaenoic acid, docosahexaenoic acid )	Wound healing	[9,23,77–79,172]
